# Supplementary material for: Analysis of PPARγ Signaling Activity in Psoriasis
Source: Int J Mol Sci. 2021 Aug 10;22(16):8603. doi: 10.3390/ijms22168603 (PMC8395241; doi:10.3390/ijms22168603)
Supplement: Supplementary file 1 [file ijms-22-08603-s001.zip › Supplemental materials_Analysis of PPARg signaling activity in psoriasis/Pathway models/Models images and html files/Model of PPARG signaling in psoriasis/749.html]

oxidized LDL


# Complex oxidized LDL

|  |  |
| --- | --- |
| URN | urn:agi-complex:oxidized%20LDL |
| Total Entities | 1 |
| Connectivity | 3943 |
| Name | oxidized LDL |

---

|  |  |
| --- | --- |
| ChildConcepts | APOB |

---

|  |  |
| --- | --- |
| Pathway | Scavenger Receptors in Platelet Activation |
|  | Scavenger Receptors in Platelet Aggregation |
|  | Macrophage M2-Related Phagocytosis |
|  | Scavenger Receptor OLR1 in Inflammation Related Endothelial Dysfunction |
|  | Dendritic Cells Function in Atherosclerosis |
|  | Low-Density Lipoproteins and Chemokines in Atherosclerosis |
|  | Androgens Synthesis in Sebocytes |
|  | Proteins Involved in Antiphospholipid Syndrome |
|  | Proteins Involved in Age-Related Macular Degeneration |
|  | ER Stress in Macular Degeneration |
|  | Oxidized LDL/OLR1 Activates Cell Proliferation in Cancer |
|  | Neighbors of oxidized LDL |
|  | Model of PPARG signaling in psoriasis |
|  | 3\_Endoplasmic reticulum stress in age-related macular degeneration |
|  | 1\_Low-density lipoprotein internalization and the accumulation of lipids in atherosclerosis |
|  | 2\_Oxidized LDL causes endothelial dysfunction in atherosclerosis |
|  | drug-target molsynthesis |

---

|  |  |
| --- | --- |
| Group | Proteins Involved in Body Weight Change Caused by Hypothyroidism |
|  | Proteins and Lipids of Drusen Associated with Age-Related Macular Degeneration |

---

|  |  |
| --- | --- |
| MedScan ID | 3000465 |

---

|  |  |
| --- | --- |
| Alias | ox-LDLs |
|  | oxLDLs |
|  | oxidized LDL |
|  | oxidized plasma LDL |
|  | ox-LDL |
|  | oxidized LDLs |
|  | oxLDL |
|  | oxidized low density lipoprotein |
|  | oxidized low density lipoproteins |
|  | oxidized plasma LDLs |

---
